# Influence of Blood Pressure Reduction on Pulse Wave Velocity in Primary Hypertension: A Meta-Analysis and Comparison With an Acute Modulation of Transmural Pressure

**DOI:** 10.1161/HYPERTENSIONAHA.123.22436

**Published:** 2024-05-09

**Authors:** Ryan J. McNally, Andrii Boguslavskyi, Rayka Malek, Christopher N. Floyd, Marina Cecelja, Abdel Douiri, Rosa-Maria Bruno, Bushra Farukh, Phil Chowienczyk, Luca Faconti

**Affiliations:** King’s College London, Department of Vascular Risk and Surgery, British Heart Foundation Centre, United Kingdom (R.J.M., C.N.F., M.C., B.F., P.C., L.F.).; Cardiac Outpatient Department, Guy’s and St. Thomas’ NHS Foundation Trust, London, United Kingdom (A.B.).; King’s College London, School of Life Course and Population Sciences, United Kingdom (R.M., A.D.).; Université Paris Cité, INSERM U970 Team 7, Paris Cardiovascular Research Centre – PARCC, France (R.-M.B.).

**Keywords:** antihypertensive agents, aorta, arterial pressure, pulse wave analysis, vascular stiffness

## Abstract

**BACKGROUND:**

Increased arterial stiffness and pulse wave velocity (PWV) of the aorta and large arteries impose adverse hemodynamic effects on the heart and other organs. Antihypertensive treatment reduces PWV, but it is unknown whether this results from an unloading of stiffer elements in the arterial wall or is due to an alternate functional or structural change that might differ according to class of antihypertensive drug.

**METHODS:**

We performed a systematic review and meta-analysis of the effects of different antihypertensive drug classes and duration of treatment on PWV with and without adjustment for change in mean arterial blood pressure (BP; study 1) and compared this to the change in PWV after an acute change in transmural pressure, simulating an acute change in BP (study 2).

**RESULTS:**

A total of 83 studies involving 6200 subjects were identified. For all drug classes combined, the reduction of PWV was 0.65 (95% CI, 0.46–0.83) m/s per 10 mm Hg reduction in mean arterial BP, a change similar to that induced by an acute change in transmural pressure in a group of hypertensive subjects. When adjusted for change in mean arterial BP, the reduction in PWV after treatment with beta-blockers or diuretics was less than that after treatment with angiotensin-converting enzyme inhibitors/angiotensin receptor antagonists or calcium channel antagonists.

**CONCLUSIONS:**

Reduction in PWV after antihypertensive treatment is largely explained by the reduction in BP, but there are some BP-independent effects. These might increase over time and contribute to better outcomes over the long term, but this remains to be demonstrated in long-term clinical trials.

**NOVELTY AND RELEVANCE**

**What Is New?**

The majority of the effect of antihypertensive treatment to reduce large artery stiffness as measured by pulse wave velocity (PWV) is accounted for by the reduction in mean arterial pressure that determines transmural pressure on the arterial wall. There is, however, some evidence of class effects with the reduction of PWV being greater for angiotensin-converting enzyme inhibitors/angiotensin II receptor blockers and calcium channel blockers compared with diuretics and beta-blockers.

**What Is Relevant?**

Irrespective of whether the reduction of PWV is caused by a reduction in mean arterial blood pressure or a specific structural effect on the arterial wall, it would be expected to have beneficial effects by ameliorating the adverse hemodynamic load imposed by arterial stiffening.

**Clinical/Pathophysiological Implications?**

Class-specific effects of antihypertensive agents to reduce PWV could increase over the long term and might be associated with better long-term outcomes, but this needs to be tested in long-term trials.

It is possible that the reduction in PWV that accompanies an acute reduction in transmural pressure could be used to predict the PWV response to a reduction in mean arterial blood pressure. This might be used as a test to identify groups or individuals in whom elevated stiffness could be effectively reduced by antihypertensive treatment.

Arterial stiffness, estimated noninvasively as carotid-femoral pulse wave velocity (PWV), predicts incident cardiovascular events,^1–5^ an association that is thought to be causally related to the hemodynamic load generated by a stiffened aorta.^6^ Thus, there has been much interest in interventions that may reduce PWV. Antihypertensive agents have consistently been shown to reduce PWV.^7,8^ However, it is not clear whether the de-stiffening is the result of structural change in the arterial wall, the mechanical effect of a reduction in the transmural pressure (TMP) distending the arterial wall that determines the distribution of load on the various components of the wall, another functional effect independent of the reduction in blood pressure (BP), or a combination of structural and functional effects.^9,10^ A specific structural effect (or functional effect) would be expected to differ according to drug class or duration of treatment.

We therefore performed a systematic review and meta-analysis of the effects of different classes of antihypertensive drugs on carotid-femoral PWV (study 1). We then conducted a physiological study examining whether the change in aortic pulse wave velocity (aPWV) with BP reduction could be accounted for by an acute reduction in TMP (study 2). We used a previously published technique to modulate TMP independent of BP^11^ by controlling the variation of intrathoracic pressure around the adventitial surface of the intrathoracic aorta. Respiratory maneuvers (Valsalva and Müller) that generate an increase or decrease in pressure around the adventitial surface of the intrathoracic aorta were used to modulate TMP and thus to simulate a change in mean arterial pressure (MAP) over a range of ≈50 mm Hg.^11^ We compared the size of change in PWV with change in MAP in interventional studies with that observed during acute modulation of TMP across the intrathoracic aorta.

## METHODS

### Data Availability

The data that support the findings of this study are available from the corresponding author upon reasonable request.

### Study 1: Meta-Analysis and Meta-Regression

#### Literature Search

A systematic review and meta-analysis were performed in accordance with PRISMA (Preferred Reporting Items for Systematic Reviews and Meta-Analyses) guidelines. PubMed, Ovid MEDLINE, Ovid Embase, and CENTRAL databases were searched from inception up to February 1, 2023, for studies investigating the effects of antihypertensive drugs on pulse wave velocity. A summary of the included studies is reported in the Supplemental Material. Case reports, letters, editorials, and reviews were excluded, and language was restricted to English. To ensure all relevant studies were included, the bibliographies of the included studies and relevant meta-analyses were screened for studies not identified by this search strategy.

#### Inclusion Criteria

Studies were included if they fulfilled the following criteria: (1) a randomized controlled trial; (2) included hypertensive subjects; (3) included antihypertensive treatment (angiotensin-converting enzyme inhibitors/angiotensin II receptor blockers (ACE inhibitors/ARB), beta-blockers (BB), calcium channel blockers (CCB), and diuretics or placebo; and (4) BP and PWV measurements at baseline and after treatment in standard format (absolute values as mean±variance). Studies were excluded if: (1) the intervention was combination antihypertensive therapy; (2) measurement of PWV was by a technique other than foot-to-foot measurements over a pathway that included the aorta; (3) nonstandard or incomplete data that did not allow calculation of change in BP and PWV after treatment; (4) interventions shorter than 4 weeks; (5) studies with <10 subjects per treatment arm; and (6) subjects with end-stage organ failure (eg, renal replacement therapy or organ transplantation).

#### Data Extraction

Data were extracted according to a predefined study protocol and included participant age, intervention (antihypertensive drug and duration of treatment), method of PWV measurement, measurement of PWV, MAP, and heart rate at baseline and postintervention. Where MAP data were not presented, they were calculated as diastolic BP+(systolic BP−diastolic BP)/3. The protocol for this systematic review was registered with PROSPERO.

#### Statistical Analyses

The primary outcome measure was the mean difference in PWV (ΔPWV; posttreatment minus baseline before treatment) for each drug class/placebo. For all analyses, *P*<0.05 was considered statistically significant. Data were analyzed using a random-effects model, with study arms weighted based on the inverse of within-study and between-study variance in ΔPWV. Each study arm was analyzed independently on the assumption that any placebo effect would be common to each study arm and that subject characteristics for the different study arms were similar. Subgroup analyses were performed to investigate the effects of different drugs on ΔPWV. Statistical analyses were conducted using Comprehensive Meta-Analysis software (version 3; Biostat).

Univariate meta-regression analyses were performed to examine the relationship between ΔPWV (dependent variable) and ΔMAP (independent variable). Multivariate regression analyses were performed to explore the effect of covariates on ΔPWV. The amount by which individual covariates explained between-study variance was quantified using a coefficient of determination (R^2^ analog). Heterogeneity was calculated using the Q statistic. Publication bias was tested by a funnel plot and Egger’s regression asymmetry test.^12^

### Study 2: Measurement of Intrathoracic Aortic PWV During Modulation of TMP by Respiratory Maneuvers

#### Subjects

Individuals (n=99) were consecutively recruited subjects attending the hypertension clinic at Guy’s and St Thomas’ Hospital. Hypertension was diagnosed on the basis of previous treatment or daytime systolic ambulatory BP (or home BP averaged ≥7 days) of >135 mm Hg systolic or >85 mm Hg diastolic, according to current guidelines.^13^ Pregnant women were excluded from the study, as were those in whom the clinical history or investigations suggested a presence of secondary hypertension. Patients with heart failure, chronic obstructive pulmonary disease, moderate or severe valvular disease, sustained nonsinus arrhythmias, or an inability to perform respiratory maneuvers were also excluded. The study was approved by the London Westminster Research Ethics Committee, and written informed consent was obtained from all patients.

#### Ultrasound Assessment of Intrathoracic Aortic PWV

Blood flow velocity was measured in the descending thoracic aorta and the abdominal aorta at the level of the diaphragm by using a pulsed-wave Doppler with 2-dimensional visualization (GE Vivid 7 ultrasound platform). The foot-to-foot time delay between Doppler waveforms at the descending aorta and abdominal aorta was determined from consecutive measurements over 3 cardiac cycles using the R wave of the ECG as a reference. Intrathoracic aPWV was calculated with path length estimated from the linear distance between the sternal notch and xiphisternum measured by a tape measure.

#### Modulation of TMP by Respiratory Maneuvers

TMP around the intrathoracic aorta was modulated by performing Valsalva and Müller maneuvers as previously described.^11^ Briefly, subjects exhaled (Valsalva) and inhaled (Müller) through a mouthpiece against a resistance with a small leak to maintain mouth pressure around +30 and −20 mm Hg for Valsalva and Müller maneuvers, respectively, for ≈15 seconds. Mouth pressure was transduced (AD Instruments, Australia) and displayed so that subjects could adjust their respiratory effort to maintain the target pressure. The mouthpiece incorporated a small leak to keep the glottis open and ensure that mouth pressure approximated intrathoracic pressure. TMP across the intrathoracic aortic wall was calculated as the difference between MAP and mouth pressure. Beat-to-beat MAP was derived from noninvasive continuous recording of arterial BP with a servo-controlled finger photoplethysmographic system (Finometer PRO; Finapres Medical Systems, the Netherlands),^14,15^ calibrated from standard oscillometric measurements (Omron, HEM-7134-E). Mouth pressure, BP, and ECG signals were simultaneously recorded using a PowerLab data acquisition system and analyzed with LabChat software (AD Instruments).

Before performing a test protocol, subjects were trained to perform Valsalva and Müller maneuvers and to maintain mouth pressure at target levels. Subjects were excluded from the study if they were unable to maintain stable mouth pressure. TMP and aPWV measured as described above were obtained during 3 Valsalva and Müller maneuvers in alternate sequence with at least 5 minutes free breathing recovery intervals (baseline) between each maneuver. Data obtained during these intervals was averaged to obtain measures of aPWV at ambient TMP (ie, TMP determined by MAP). Repeated measurements of aPWV and TMP during baseline and respiratory maneuvers were averaged for every subject. The mean values of aPWV and TMP were then calculated at baseline and Valsalva and Müller maneuvers and linear regression analysis were used to calculate the slope of the relationship between aPWV and TMP.

## RESULTS

### Study 1: Meta-Analysis and Meta-Regression

#### Study Selection

**Figure 1.**
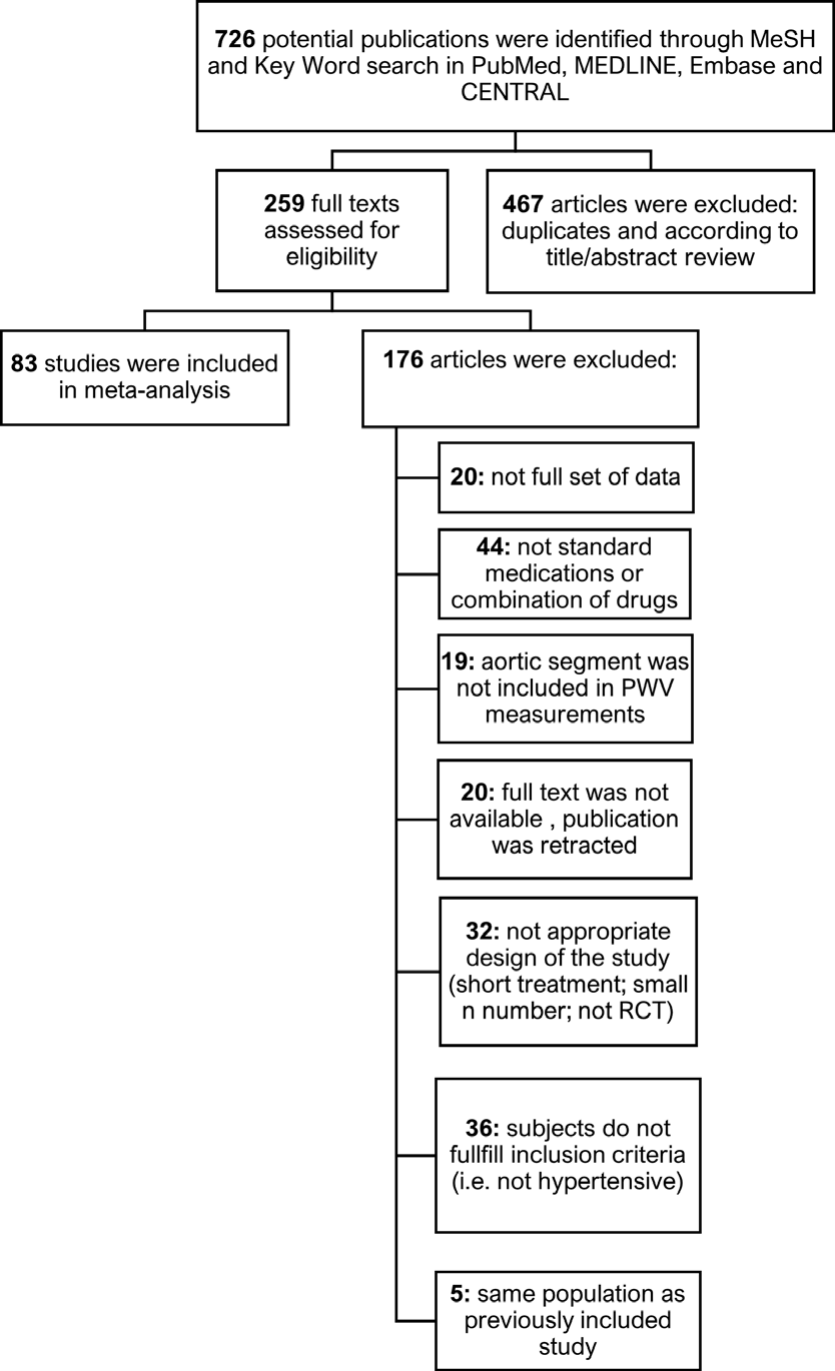
**Flow chart shows inclusion and exclusion of studies in the systematic review.** PWV indicates pulse wave velocity; and RCT, randomized controlled trial.

In all, 727 articles were identified by the literature search, of which 83 studies containing 6200 subjects met our inclusion criteria (Figure 1). A total of 16 studies used placebo, 58 ACE inhibitors/ARB, 30 BB, 32 CCB, and 20 diuretics (12 thiazide/thiazide-like diuretics and 8 mineralocorticoid receptor antagonists). The mean±SD participant age was 57.51±8.2 years, and the median duration of treatment was 12.5 weeks (interquartile range, 10–26 weeks). The most common method of measurement of PWV was carotid-femoral PWV using the SphygmoCor or Complior devices (56/83 studies) with other measurements of aortic PWV (3/83) and brachial-ankle measurements in the remainder (24/83).

#### Overall Analysis Based on Drug Class

**Figure 2.**
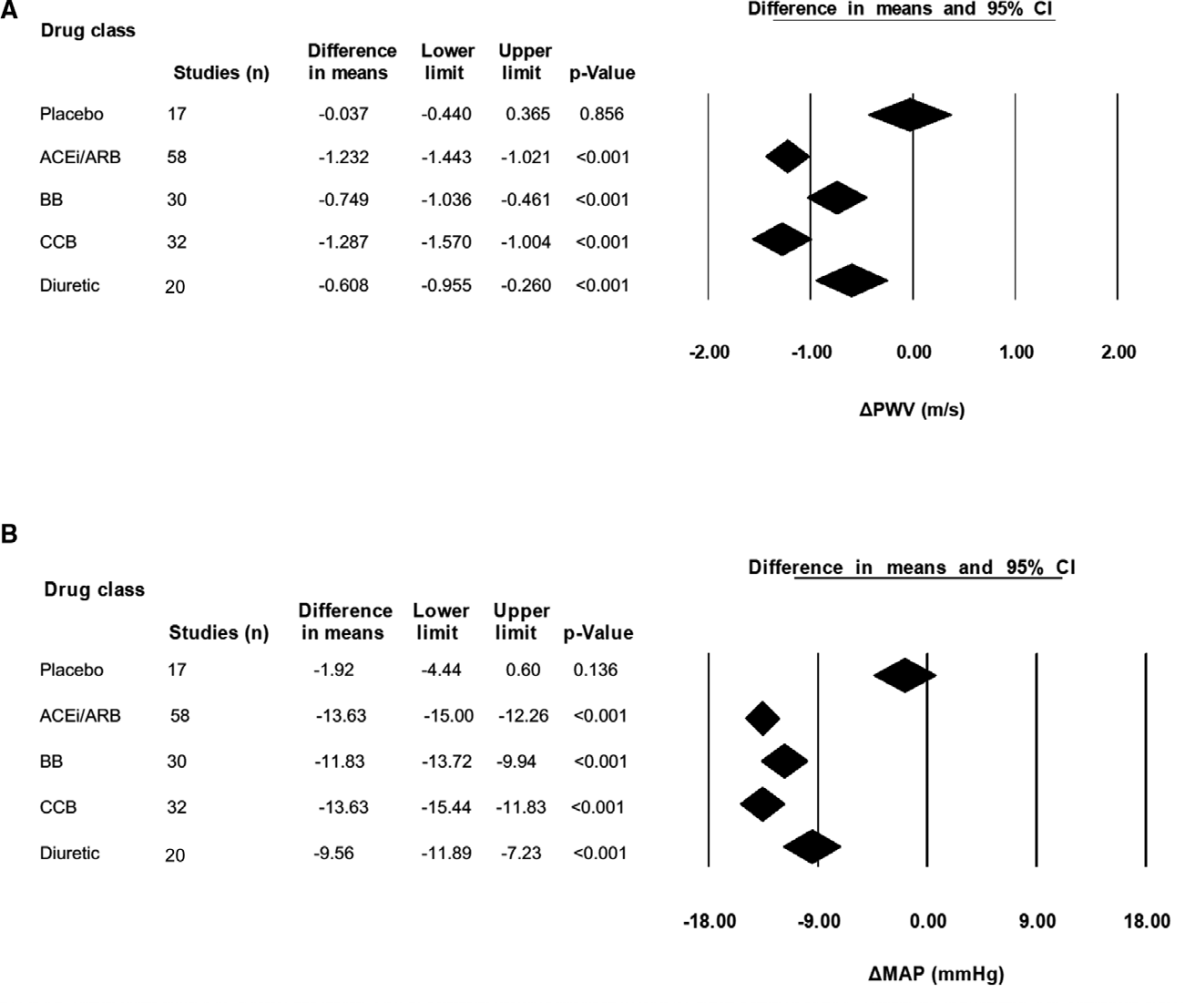
**Change in pulse wave velocity (ΔPWV) and mean arterial pressure (ΔMAP) in response to placebo or antihypertensive drugs. A**, PWV. **B**, MAP. ACEi/ARB indicates angiotensin-converting enzyme inhibitors/angiotensin II receptor blockers; BB, beta blocker; and CCB, calcium channel blocker.

All antihypertensive drug classes resulted in a significant decrease in PWV, as shown in Figure 2A. The magnitude of this varied significantly between drug classes (Q=14.38; *P*=0.002), with the largest decrease observed for ACE inhibitors/ARB (−1.23 [95% CI, −1.4 to −1.0] m/s; *P*<0.001; with no significant difference between ACE inhibitors and ARB) and the smallest for diuretics (−0.62 [95% CI, −0.97 to −0.27] m/s; *P*<0.001).

A significant decrease in MAP was also observed for each drug class (Figure 2B) with significant heterogeneity between the 4 drug classes (Q=8.68; *P*=0.034), with the largest decrease in MAP observed for CCB (−13.9 [95% CI, –15.44 to −11.83] mm Hg; *P*<0.001) and the smallest for diuretics (−9.88 [95% CI, −12.25 to −7.49] mm Hg; *P*<0.001).

**Figure 3.**
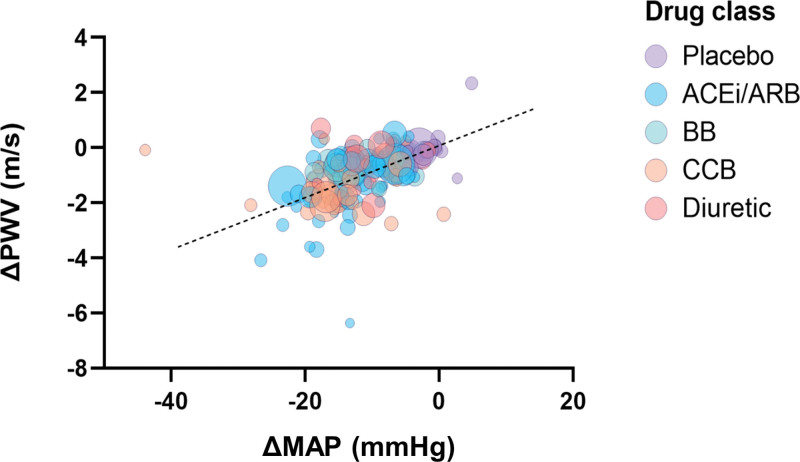
**Meta-regression of change in pulse wave velocity (ΔPWV) versus change in mean arterial pressure (ΔMAP) during placebo and antihypertensive drug treatment.** Bubble size is representative of sample size. ACEi/ARB indicates angiotensin-converting enzyme inhibitors/angiotensin II receptor blockers; BB, beta blocker; and CCB, calcium channel blocker. Y=0.065x–0.19 (95% CI, 0.047–0.083; *P*<0.001).

Meta-regression revealed that reductions in PWV and MAP were significantly associated (regression coefficient 0.0648 [95% CI, 0.047–0.083]; *P*<0.001; Figure 3). Inclusion of other covariates: age, baseline MAP, baseline PWV, and study duration in multivariate meta-regression had little influence on the correlation of reduction in PWV with that of MAP (coefficient 0.0626 [95% CI, 0.036–0.089] m/s; *P*<0.001).

**Figure 4.**
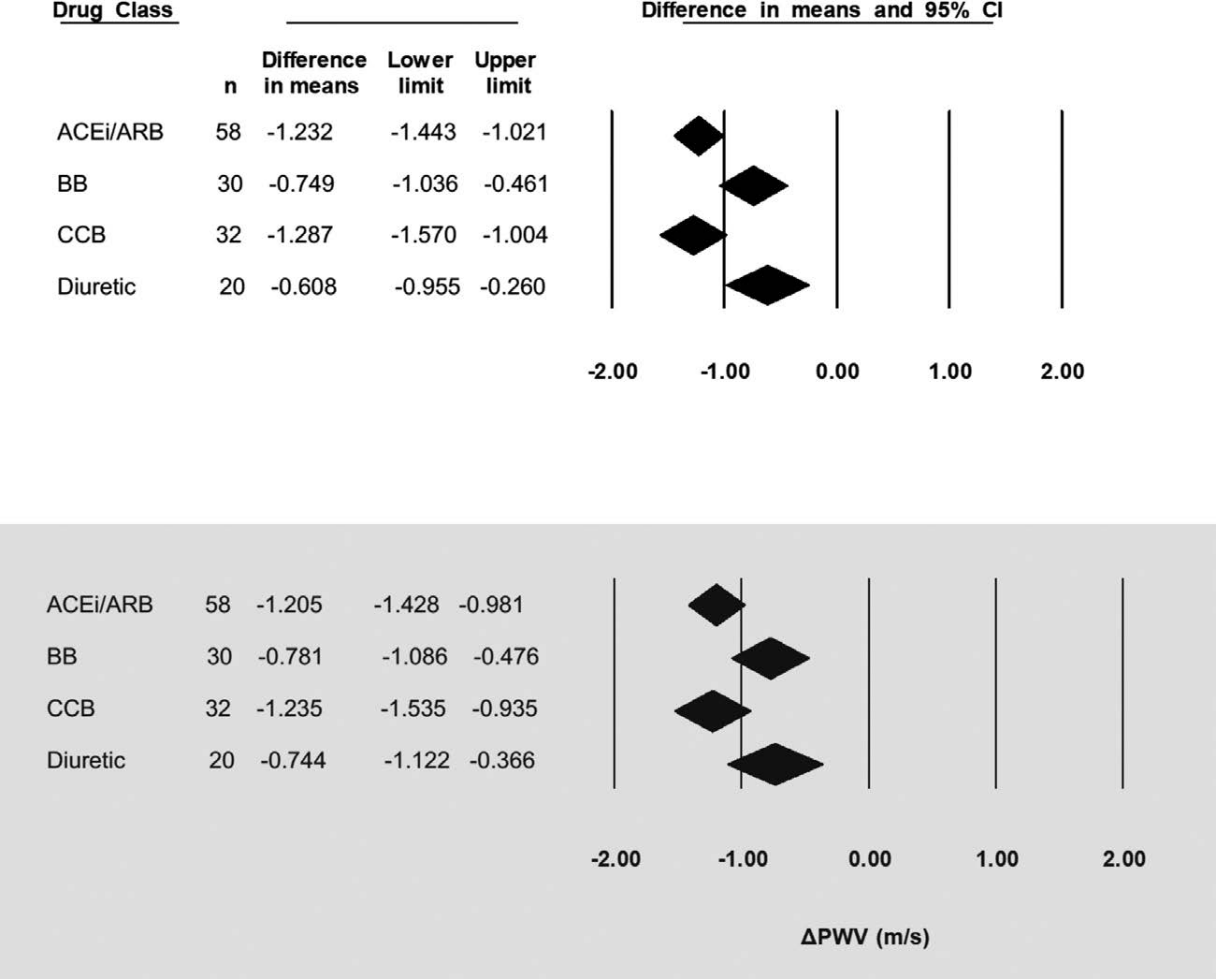
**Change in pulse wave velocity in response to antihypertensive drugs. Top** panel is before adjustment for change in mean arterial pressure (ΔMAP; mm Hg). **Bottom** panel is for adjusted for ΔMAP (mm Hg). ΔPWV indicates change in pulse wave velocity; ACEi/ARB, angiotensin-converting enzyme inhibitors/angiotensin II receptor blockers; BB, beta blocker; and CCB, calcium channel blocker.

After adjustment for change in MAP, PWV decreased by the greatest amount for CCB and ACE inhibitors/ARB (−1.24 [95% CI, −1.54 to −0.94] m/s and −1.21 [95% CI, −1.43 to −0.98] m/s, respectively) and to a lesser extent with BB and diuretics (−0.78 [95% CI, −1.09 to −0.48] m/s and −0.74 [95% CI, −1.12 to −0.37] m/s, respectively), within significant between-class differences (*P*<0.05; Figure 4).

**Figure 5.**
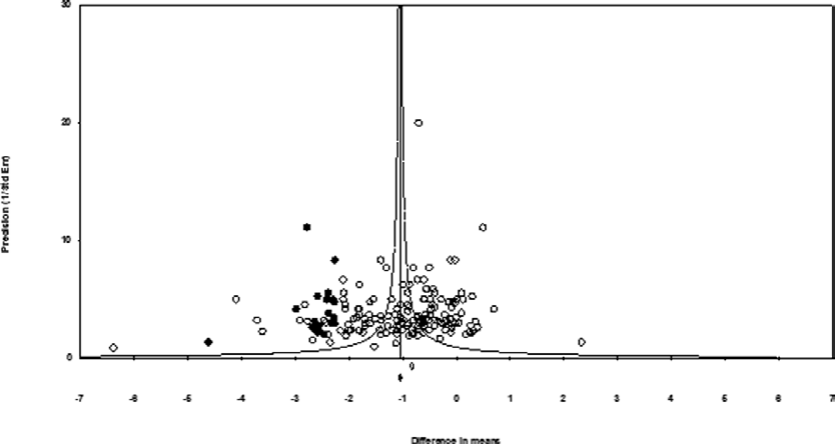
**Funnel plot to assess publication bias.** For each study, the difference in means for change in pulse wave velocity is shown against study precision. The open diamond below the *x* axis indicates the pooled effect size; closed diamond represents imputed effect size.

The funnel plot of SE versus effect size (ΔPWV) was asymmetrical and suggestive of potential publication bias. The presence of publication bias was also suggested by Egger’s linear regression (*P*=0.028). After adjustment of effect size for potential publication bias using the trim and fill correction, 21 potentially missing studies on the left side of the funnel plot were imputed, leading to a corrected overall effect size of −1.05 [95% CI, −1.08 to −1.01] m/s in PWV; Figure 5).

### Study 2: Influence of Acute Modulation of TMP on PWV

**Figure 6.**
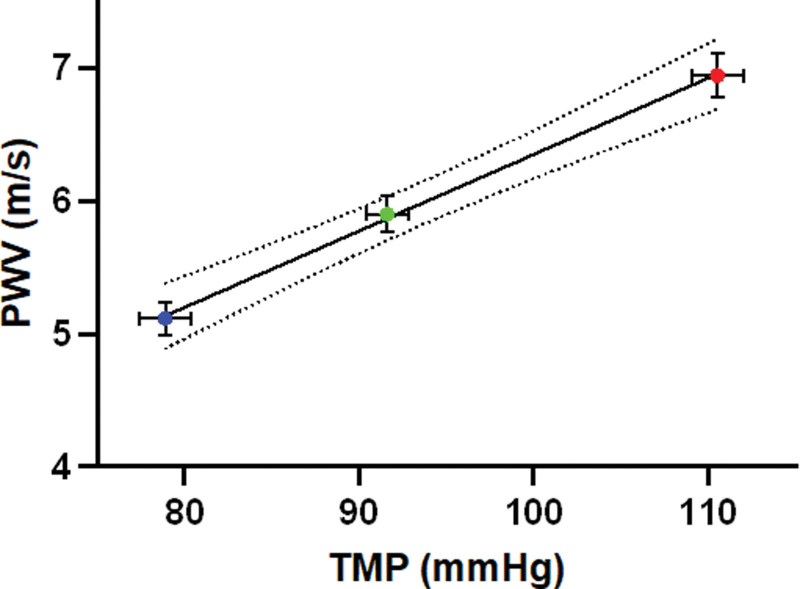
**Variation of pulse wave velocity (PWV) with transmural pressure (TMP) during free breathing (baseline), expiration (Valsalva) and inspiration (Müller).** Error bars represent SEM, n=99. Y=0.058x+0.59.

The influence of an acute change in TMP on PWV was studied in 99 subjects. Participants were predominantly young to middle-aged (mean age, 41.6±14.3 years) and women (52%) of White background with the majority being nonsmokers (89%). Average body mass index was 24.7±3.45 kg/m^2^ and total cholesterol 5.16±1.01 mmol/L. Baseline MAP and aPWV were 91.6±11.8 mm Hg and 5.90±1.38 m/s, respectively. Changes in aPWV (Figure 6) were strongly related to those of TMP with a change in PWV of 0.58 m/s per 10 mm Hg and a change in TMP (95% CI, 0.45–0.7 m/s per 10 mm Hg; *P*<0.0001).

## DISCUSSION

An association of PWV with BP is an almost universal finding in epidemiological studies.^16^ An acute effect of a change in MAP to produce a parallel change in PWV in experimental models and in vivo in humans is also well-recognized.^11,17^ It is explained because BP determines TMP tending to stretch the arterial wall and will alter how load on the arterial wall is distributed between elastic and stiffer elements of the wall (elastin and collagen, respectively). An increase in pressure and stretch transfers load to stiffer collagen elements, leading to greater arterial stiffness.^18^ To what extent the association of PWV with hypertension is due simply to the mechanical effects of high ambient BP on wall mechanics or to the longer-term effects of hypertension inducing structural change in the arterial wall has been examined in several experimental studies, and the results have been conflicting.^11,17,19,20^ Current evidence points to the importance of both effects with structural change dependent on long-term mechanical insults of hypertension together with other age-related degenerative processes.^21^

Because age-related increases in PWV drive systolic hypertension, impose other adverse hemodynamic effects, and are predictive of incident cardiovascular events, there has been intense interest in interventions that may reduce PWV.^22^ Antihypertensive treatment reduces PWV, but the extent to which this is a pure hemodynamic effect due to a reverse of the loading phenomenon associated with increased BP or reflects structural or functional alterations that are independent of BP is unknown. Structural change in the arterial wall could be prevented either by action on a specific pathway (eg, antifibrotic agents that block the renin-angiotensin-aldosterone pathway) or by any BP-lowering treatment, irrespective of mechanism. The former effect would be expected to be restricted to certain drug classes, whereas the latter could be independent of drug class but would be expected to be dependent on the duration of treatment. In the present study, we investigated to what extent a reduction in PWV in response to either antihypertensive medications or physiological maneuvers was determined by the corresponding reduction in BP (MAP).

The meta-analysis and meta-regression showed that all antihypertensive drug classes reduce both PWV and MAP, and there is a significant correlation between the reduction of PWV and that of MAP. This change in PWV with a change in MAP was similar to that observed when a change in MAP was simulated by an acute reduction in TMP acting to distend the arterial wall. Comparison between these studies is, however, limited by the different vascular segments studied (thoracic aorta in our physiological study of acute modulation of TMP and carotid-femoral pathway in the majority of studies in the meta-analysis), the possibility that the breathing maneuvers used to change TMP also impacted PWV through modulation of the autonomic nervous system,^23^ and the differing characteristics of the individuals in the physiological study on acute modulation of TMP compared with those in the meta-analysis. Nevertheless, these data are consistent with much of the effects of antihypertensive agents being due to the reduction in BP per se rather than by any specific effect of the drugs on the arterial wall. When adjustments for change in MAP were made to enable comparison of the reduction in PWV for the same reduction in MAP, there were differences between drug classes that reached statistical significance, with ACE inhibitors/ARB and CCB having a greater effect on reducing PWV than BB and diuretics. It is notable that similar results have been found in another analysis,^24^ where ACE inhibitors had the greatest effect on PWV reduction when adjusted for change in MAP. A drug-specific effect on arterial stiffness could theoretically contribute to the more favorable outcomes associated with ACE inhibitor/CCB treatment compared with BB/diuretic treatment.^25^

Our analysis would suggest, therefore, that antihypertensive drugs reduce PWV both by a nonspecific effect in reducing ambient BP, thus reducing distending forces on the arterial, and by effects that may be specific to certain antihypertensive drug classes. Irrespective of the mechanism of de-stiffening, reduction of PWV is likely to have beneficial effects beyond that of concomitant reduction in MAP, with a reduction in pulse pressure (which is in part determined by PWV), on dynamic load on the heart, and reduction of transmission of pulsatile forces to the microcirculation in the brain and kidneys.^26^ Indeed, the BP dependence of PWV may explain why drugs that act predominantly on resistance vessels to reduce MAP also reduce pulse pressure and systolic BP. The reduction of MAP leads, in fact, to a reduction in PWV and a reduction in pulse pressure.

Our study is subject to several limitations common to meta-analyses in terms of publication bias and heterogeneity. Although we attempted to adjust for publication bias, it is possible that this favored newer agents such as ACE inhibitor/ARB. We analyzed individual treatment arms of studies independently on the assumption that any placebo effect would be common to all classes of drugs. A small (and statistically nonsignificant) placebo effect was observed in relation to a reduction in MAP that may have magnified the strength of the relationship between the change in PWV and that of MAP. Regression toward the mean may also have increased the magnitude of this relationship. Background treatment could have had a confounding effect, and we cannot exclude the effect of confounding factors resulting from some imbalance in subject characteristics between the study arms. The comparison of the acute physiological study to modulate TMP with the results of the meta-analysis is subject to the limitations previously discussed.

Finally, the studies we examined were of relatively short duration (median, 12.5 weeks), and we cannot exclude a BP-independent remodeling effect over a longer period. Although 12 weeks is considered long enough to induce structural change, previous studies have shown that much longer treatments may be necessary to show true pressure-independent changes in stiffness,^27^ and it is possible that the BP-independent effects that we observed in the present study may become more pronounced with a longer duration of treatment. Most hypertensive patients are treated with >1 antihypertensive drug, and our study did not address the effect of drug combinations on PWV. Similarly, we did address differences relating to sex or ethnicity, which would require individual patient data.

### Perspectives

Given the prognostic importance of PWV, it is important to determine whether antihypertensive treatments might have a specific effect on reducing PWV or whether such effects are mediated simply through a reduction in the BP acting to distend the arterial wall. The present study suggests that while the majority of the effect is mediated through a reduction in BP, there are some class-specific effects that are likely to relate to a structural or functional change in the arterial wall that is independent of the change in BP. ACE inhibitor/ARB and CCB drug classes appear to be more effective than BB and diuretics after adjustment for change in MAP. This class-specific effect on PWV could contribute to the better outcomes associated with the use of these classes of drugs, although many other factors could also be responsible. Even when effects are due to a reduction in BP alone, they are likely to provide hemodynamic benefit and reduce hypertension-mediated organ damage. The reduction of PWV for a given degree of reduction in BP might be predicted from the pressure-dependence of PWV measured during acute modulation of TMP, but further studies will be required to test this hypothesis.

In conclusion, the reduction in PWV after antihypertensive treatment is largely explained by the concomitant reduction in BP. There are some BP-independent effects that might contribute to better outcomes over the long term, but this remains to be demonstrated in long-term clinical trials.

## ARTICLE INFORMATION

### Sources of Funding

This study was supported by a stratified medicines grant from the Medical Research Council: MR/M016560/1.

### Disclosures

None.

## Supplementary Material


