# Supplementary material for: Influence of Blood Pressure Reduction on Pulse Wave Velocity in Primary Hypertension: A Meta-Analysis and Comparison With an Acute Modulation of Transmural Pressure
Source: Hypertension. 2024 May 9;81(7):1619–27. doi: 10.1161/HYPERTENSIONAHA.123.22436 (PMC11177599; doi:10.1161/HYPERTENSIONAHA.123.22436)
Supplement: Supplementary file 1 [file hyp-81-1619-s001.docx]

**Influence of blood pressure reduction on pulse wave velocity in primary hypertension: a meta- analysis and comparison with an acute modulation of transmural pressure**

**Supplemental Material**

Ryan John McNally^1^, PhD* [ryan.mcnally@kcl.ac.uk](mailto:ryan.mcnally@kcl.ac.uk)

Andrii Boguslavskyi^2^, PhD*, andrii.boguslavskyi@kcl.ac.uk

Rayka Malek^3^, MSc, rayka.malek@kcl.ac.uk

Christopher N Floyd^1^, PhD, christopher.floyd@kcl.ac.uk

Marina Cecelja^1^, PhD, marina.3.cecelja@kcl.ac.uk

Abdel Douiri^3^, PhD, abdel.douiri@kcl.ac.uk

Rosa-Maria Bruno^4^, PhD, rosa-maria.bruno@inserm.fr

Bushra Farukh, MSc^1^, bushra.farukh@kcl.ac.uk

Phil Chowienczyk^1^, FRCP^^^, phil.chowienczyk@kcl.ac.uk

Luca Faconti^1^,PhD^^^ [luca.faconti@kcl.ac.uk](mailto:luca.faconti@kcl.ac.uk)

1. King’s College London, Department of Vascular Risk and Surgery, British Heart Foundation Centre, London, UK
2. Cardiac Outpatient Department, Guy’s and St. Thomas’ NHS Foundation Trust, London, UK
3. King’s College London, School of Life Course and Population Sciences, London, UK
4. Université Paris Cité, INSERM U970 Team 7, Paris Cardiovascular Research Centre – PARCC, Paris, France

*Joint first author ^Joint senior author

Correspondence:

Phil Chowienczyk, Department of Clinical Pharmacology, St Thomas’ Hospital, London, UK, SE1 7EH; Tel: 0044-2071884799; Fax: 0044-2071885116;

Email: phil.chowienczyk@kcl.ac.uk

**Table S1: Individual studies in systematic review of change in pulse wave velocity (PWV) with change in mean arterial blood pressure (MAP)**

| Author | Year | Place of study, country | N (% Female) | Age, years | Co-morbidity | PWV method | Intervention Arms | Duration, weeks |
| --- | --- | --- | --- | --- | --- | --- | --- | --- |
| Agnoletti, D. | 2013 | Asia/Europe | 145 (47.6) | 58.2 | - | Car-Fem | Indapamide; Candesartan; Amlodipine; Placebo | 12 |
| Eguchi, K. | 2015 | Asia: Japan | 99 (33.7) | 59.0 | - | Br.-Ankle | Celiprolol;  Bisoprolol. | 12 |
| Frimodt-Moller, M. | 2012 | Europe | 57 (22.4) | 60.0 | CKD | Car-Fem | Enalapril/Candesartan. | 24 |
| Georgianos, P. | 2015 | USA | 109 (33.5) | 52.1 | CKD | Aortic | Atenolol;  Lisinopril | 24 |
| Hayek, S. S. | 2015 | USA | 30 (43) | 55.0 | - | Car-Fem | Metoprolol;  Nebivolol. | 26 |
| Laurent, S. | 2014 | Europe | 116 (37.1) | 52.5 | Metab. syndrome | Car-Fem | Olmesartan 20mg; 40mg; 80mg. | 52 |
| Nakamura, T. | 2011 | Asia: Japan | 35 (32.9) | 57.8 | - | Br.-Ankle | Placebo | 8 |
| Raff, U. | 2015 | Europe: Germany | 69 (31.9) | 51.5 | Metab. syndrome | Car-Fem | Omlesartan 20mg; 80mg;  Amlodipine. | 6 |
| Ali, K. | 2009 | Europe: UK | 15 (-) | 65.1 | - | Car-Fem | Irbesartan;  Lisinopril. | 12 |
| Asmar, R. | 1991 | Europe: France | 14 (50) | 53.0 | - | Car-Fem | Bisoprolol  Placebo | 4 |
| Asmar, R. | 2001 | Europe/ Australia | 170 (-) | 51.0 | - | Aortic PWV | Atenolol. | 52 |
| Dhakam, Z. | 2006 | Europe: UK | 21 (38.1) | 51.0 | - | Car-Fem | Atenolol;  Eprosartan. | 6 |
| Dhakam, Z. | 2008 | Europe: UK | 16 (37.5) | 70.0 | - | Car-Fem | Atenolol;  Nebivolol;  Placebo | 5 |
| Hayoz, D. | 2012 | Europe: Switzerland | 109 (100) | 61.0 | - | Car-Fem | Valsartan;  Amlodipine. | 38 |
| Ichihara, A. | 2006 | Asia: Japan | 100 (26) | 54.0 | - | Br.-Ankle | Amlodipine;  Valsartan. | 52 |
| Jin, Y. | 2011 | Europe/ Australia | 94 (31.3) | 55.0 | - | Car-Fem | Atenolol. | 52 |
| Kampus, P. | 2011 | Europe: Germany | 63 (48.8) | 46.0 | - | Car-Fem | Nebivolol;  Metoprolol. | 52 |
| Karalliedde, J. | 2008 | USA | 53 (41.2) | 60.0 | T2DM | Car-Fem | Amlodipine | 24 |
| Kithas, P. A. | 2010 | USA | 45 (44.4) | 69.5 | - | Car-Fem | HCTZ;  Spironolactone. | 26 |
| Kosch, M. | 2008 | Europe: Germany | 52 (45.6) | 45.8 | - | Car-Fem | Valsartan;  Metoprolol. | 13 |
| Koumaras, C. | 2014 | Europe: Greece | 54 (31.9) | 47.0 | - | Car-Fem | Quinapril;  Atenolol;  Nebivolol. | 10 |
| Mackenzie, I. S. | 2009 | Europe: UK | 59 (47.5) | 68.5 | - | Car-Fem | Perindopril;  Atenolol; Lercanidipine;  Bendrofluazide. | 10 |
| Matsui, Y. | 2011 | Asia: Japan | 207 (59.9) | 68.0 | - | Car-Fem | Azelnidipine;  HCTZ | 24 |
| Morimoto, S. | 2006 | Asia: Japan | 43 (58.1) | 57.0 | - | Br.-Ankle | Amlodipine;  Telmisartan. | 24 |
| Munakata, M. | 2004 | Asia: Japan | 41 (51.2) | 54.0 | - | Br.-Ankle | Valsartan;  Nifedipine | 12 |
| Rajzer, M. | 2003 | Europe: Poland | 99 (54.2) | 54.0 | - | Car-Fem | Quinapril;  Amlodipine;  losartan | 26 |
| Rhee, M. Y. | 2011 | Asia: Korea | 34 (56.3) | 56.5 | - | Br.-Ankle | Placebo | 13 |
| Spanos, G. | 2013 | Europe: Greece | 14 (31) | 57.5 | CKD | Car-Fem | Valsartan. | 24 |
| Takami, T. | 2003 | Asia: Japan | 76 (0) | 71.5 | - | Br.-Ankle | Valsartan;  Temocapril;  Cilnidipine;  Nifedipine. | 13 |
| Tomiyama, H. | 2011 | Asia: Japan | 113 (36.3) | 57.0 | - | Br.-Ankle | Candesartan;  Amlodipine. | 104 |
| Safar, M. E. | 1991 | Europe: France | 13 (23.1) | 48.0 | - | Car-Fem | Verapamil;  Placebo. | 12 |
| Park, S. | 2013 | Asia: Korea | 191 (39.3) | 52 | - | Car-Fem | Bisoprolol;  Atenolol. | 12 |
| Ichihara, A. | 2007 | Asia: Japan | 50 (44) | 61 | - | Br.-Ankle | Amlodipine;  Placebo. | 52 |
| Kaneshiro, Y. | 2009 | Asia: Japan | 68 (27.9) | 53 | CKD | Br.-Ankle | Amlodipine;  HCTZ. | 52 |
| Mahmud, A. | 2005 | Europe: Ireland | 24 (41.7) | 50 | - | Car-Fem | Spironolactone; Bendroflumetazide. | 4 |
| Pathapati, R. | 2015 | S. Asia: India | 60 (25) | 50.5 | - | Car-Fem | Amlodipine;  Cilnidipine. | 8 |
| Ishii, H. | 2008 | Asia: Japan |  | 68 | DII | Br.-Ankle | Candesartan. | 12 |
| Nakayama, T. | 2007 | Asia: Japan | 40 (45) | 72.5 | Cerebral infarction | Heart-Ankle | Telmisartan;  Placebo. | 12 |
| Mahmud, A. | 2008 | Europe: Ireland | 40 (42.5) | 47.5 | - | Car-Fem | Atenolol ;  Nebivolol. | 4 |
| Mitchell, G. | 2005 | Canada/USA | 84 (34.5) | 61 | - | Car-Fem | Enalapril. | 12 |
| Topouchian, J. | 1999 | Europe: France | 46 (45.7) | 53 | - | Car-Fem | Verapamil;  Trandolapril. | 24 |
| Aparicio, L. S. | 2015 | Argentina | 19 (52.6) | 45 | - | Car-Fem | Atenolol;  Bisoprolol. | 4 |
| Moltzer, E. | 2010 | Europe: Netherlands | 16 (37.5) | 37 | Aortic coarctation | Car-Fem | Candesartan; Metoprolol. | 8 |
| Nakamura, T. | 2008 | Asia: Japan | 30 (40) | 46 | CKD | Br.-Ankle | Amlodipine; Telmisartan. | 52 |
| Kapil, V. | 2015 | Europe: UK | 32 (68.8) | 56 | - | Car-Fem | Placebo. | 4 |
| Mizuno, H. | 2016 | Asia: Japan | 52 (69.2) | 77 | - | Br.-Ankle | Amlodipine. | 16 |
| Mahmud, A. | 2002 | Europe: Ireland | 11 (54.5) | 57 | - | Car-Fem | Losartan;  HCTZ. | 4 |
| Ziegler, M. | 2017 | USA | 31 (19.4) | 52 | Apnea-hypopnea | Car-Fem | Nebivolol;  HCTZ. | 6 |
| Edwards, N. | 2009 | Europe: UK | 112 (42) | 53.5 | CKD | Car-Fem | Spironolactone; Placebo. | 40 |
| Ohta, Y | 2015 | Asia: Japan | 20 (45) | 71 | - | Br.-Ankle | Eplerenone; Indapamide. | 12 |
| Briasoulis, A. | 2013 | USA | 61 (70.5) | 65 | DII | Car-Fem | Nebivolol;  Metoprolol. | 26 |
| Williams, B. | 2017 | Europe, South America, Asia, USA | 225 (47.6) | 67 | - | Car-Fem | Olmesartan. | 12 |
| White, W. | 2003 | Canada/USA | 269 (52.8) | 68 | - | Car-Fem | Eplerenone;  Amlodipine. | 24 |
| Anan, F. | 2005 | Asia: Japan | 21 (52.4) | 59 | DII | Br.-Ankle | Valsartan;  Perindopril. | 40 |
| Posadzy-Malaczynska, A. | 2015 | Europe: Poland | 100 (100) | 51 | - | Car-Fem | HCTZ;  Perindopril. | 52 |
| London, G. | 2004 | Europe (France), Australia | 93 (23.7) | 57.5 | - | Car-Fem | Atenolol. | 52 |
| Cunha, A. | 2017 | Brazil | 18 (100) | 57 | - | Car-Fem | Placebo. | 24 |
| Nedogoda, S. | 2013 | Europe: Russia | 120 (49.2) | 48 | Obese | Car-Fem | Perindopril;  Enalapril;  Losartan;  Telmisartan. | 24 |
| Mahmud, A. | 2002 | Europe: Ireland |  | 49 | - | Car-Fem | Valsartan;  Captopril. | 4 |
| Komai, N. | 2002 | Asia: Japan | 25 (36) | 61.5 | - | Car-Fem | Cilazapril;  Atenolol. | 24 |
| Hong, S. | 2011 | Asia: Korea | 73 (24.7) | 62.5 | DII | Br.-Ankle | Telmisartan;  Valsartan. | 32 |
| Lim, S. | 2011 | Asia: Korea | 60 (36.7) | 48.5 | - | Car-Fem | Telmisartan;  Valsartan. | 12 |
| Park, J. | 2015 | USA | 32 (0) | 55 | CKD | Car-Fem | Placebo | 12 |
| Liu, Q. | 2016 | Asia: China | 386 (48.4) | 71.5 | - | Br.-Ankle | Olmesartan;  Placebo | 12 |
| Virdis, A. | 2012 | Europe: Italy | 25 (32) | 45 | - | Car-Fem | Ramipril. | 12 |
| Masuda, S. | 2009 | Asia: Japan | 30 (40) | 61 | DII, CKD | Br.-Ankle | Losartan;  Telmisartan. | 12 |
| Dhaun, N. | 2011 | Europe: UK | 27 (14.8) | 48 | CKD | Car-Fem | Placebo;  Nifedipine. | 6 |
| Eguchi, K. | 2016 | Asia: Japan | 57 (36.8) | 63 | - | Br.-Ankle | Eplerenone;  Placebo. | 12 |
| Morimoto, S. | 2008 | Asia: Japan | 32 (53.1) | 64 | - | Br.-Ankle | Amlodipine; Perindopril. | 24 |
| Takami, T. | 2011 | Asia: Japan | 50 (30) | 67 | - | Br.-Ankle | Azelnidipine; Amlodipine. | 24 |
| Asmar, R | 1993 | Europe: France | 16 (31.3) | 53 | - | Car-Fem | Felodipine;  HCTZ | 6 |
| Mitchell, G | 2002 | USA | 87 (35.6) | 61 | - | Car-Fem | Enalapril;  Omapatrilat | 12 |
| Shao-Kun, X | 2019 | Asia: China | 36 (42.9) | 53.8 | - | Br.-Ankle | Nifedipine | 12 |
| Zhang, G | 2020 | Asia: China | 16 (25.4) | 42.7 | - | Br-Ankle | Allisartan | 4.2 |
| Ramirez, A | 2019 | Argentina | 5 (13.2) | 59 | - | Car-Fem | Perindopril | 24 |
| Rogers, S | 2022 | USA | 68 (55) | 65 | - | Car-Fem | Candesartan, Lisinopril | 52 |
| Zhang, J | 2019 | Asia: China | 32 (50) | 65 | - | Br-Ankle | Allisartan, Nifedipine | 24 |
| Dudinskaya, E | 2021 | Russia | 46 (100) | 61.61 | - | Car-Fem | Bisoprolol | 52 |
| Oliveras, A | 2018 | Europe: Spain | 4 (31) | 64.9 | - | Car-Fem | Spironolactone | 24 |
| Liu, Y | 2018 | Asia: China | 79 (42) | 55.6 | - | Car-Fem | HCTZ, Spironolactone | 4 |
| Schmieder, R | 2017 | Europe:  Germany | 17 (29.8) | 59.2 | - | Car-Fem | Olmesartan | 12 |
| Wang, Y | 2020 | Asia: China | 151 (63) | 59.3 | - | Br-Ankle | Lacidipine, Amlodipine | 20 |
| Shanmugarajan, D | 2020 | Asia: India | 18 (24) | 60 | - | Car-Fem | Placebo | 12 |
